# Spinal Cord Image Denoising Using Dncnn Algorithm

**DOI:** 10.2174/0115734056337613241209072322

**Published:** 2025-02-28

**Authors:** R. Jerlin, Priya Murugasen, N.R Shanker

**Affiliations:** 1 Department of ECE, Anna University, Chennai, India; 2 Department of Physics, Saveetha Engineering College, Thandalam, Chennai 602 105, India; 3 Department of CSE, Aalim Muhammed Salegh College of Engineering, Chennai, India

**Keywords:** MR spine image, Denoising convolutional neural network (DnCNN), Discrete wavelet transform (DWT), Stationary wavelet transform (SWT), Nucleus pulposus, Vertebrae

## Abstract

**Background:**

Spinal image denoising plays a vital role in the accurate diagnosis of disc herniation (DH).

**Objective:**

Traditional denoising algorithms perform less due Limited Directional Selectivity problem and do not adequately capture directional information in pixels. Traditional algorithms' edge representation and texture details are insufficient for the earlier detection of DH. Limited Directional Selectivity leads to inaccurate diagnosis and classification of Disc Herniation (DH) stages. The DH stages are (i) Degeneration (ii) Prolapse (iii) Extrusion and (iv) Sequestration. Moreover, detection of DH size below 2mm using MR image is the major problem.

**Methods:**

To solve the above problem, spinal cord MR images fed to the proposed Parrot optimization tuned Denoising Convolutional Neural Network (Po-DnCNN) algorithm for perspective enhancement of nucleus pulposus region in the spinal cord, vertebrae. The perspective enhancement of Spinal cord image led to the accurate classification of stages and earlier detection of DH by using the proposed Hippopotamus optimization- Fast Hybrid Vision Transformer (Ho–FastViT) algorithm. For this study, spinal cord MR images are obtained from the Grand Challenge website – SPIDER dataset.

**Results:**

The proposed Po-DnCNN method and Ho-FastViT results are analysed quantitatively and qualitatively based on the edge, contrast, classification of the stage, and enhancement of the projected nucleus pulposus region in the spinal cord and vertebrae. The predicted DH results using the proposed method are compared with the manual Pfirrman Grade value of the spinal card method.

**Conclusion:**

Proposed method is better than traditional methods for earlier detection of DH. Po-DnCNN and Ho-FastViat methods give high accuracy of about 98% and 97% compared to traditional methods.

## 1 INTRODUCTION

The spinal column or vertebral column is connected through a bunch of muscles, bones, and joints. Vertebral column consists of 33 vertebrae in serial. The spinal column provides the structure of the human body. The central hollow position of vertebrae is the nerve canal. Spinal canal moves down the length of the spine and is surrounded by the spinal cord. The vertebral column is classified into five types such as cervical spine (C1-C7), thoracic spine (T1-T12), lumbar spine (L1-L5), sacral spine (S1-S5) and tailbone. Each vertebra is segregated through the disc. The cervical spine is at the neck, which begins at the base of the skull. The first cervical vertebrae of ring-shaped bone are called as atlas. Cervical vertebra protects the spinal cord and allows head movement and balances the head. Thoracic vertebrae are located at the ribs and sternum. Lumbar vertebras are located between the thoracic and sacral vertebrae. Disc is a spongy cushion, which separates the vertebrae. Discs allow the vertebrae movement through pivot points and absorb the shock and keep the spine in stable condition. An intervertebral fibro cartilage of an intervertebral disc lies between adjacent vertebrae in a vertebral column. Disc has interior and exterior portions. The interior portion is soft and called the nucleus pulpous. Exterior portion is tough and called annulus fibrosus. This exterior portion is composed of a ring of ligament fibers.

Disc problems are worn out, ligaments stretch, and nerves squeeze leading to pain. In old age, the disc has less water content, becomes dry and decreases sponginess, which leads to reduced shock absorption. The outer layer causes disc bulge and compression of the nerve in the spine. The symptoms of disc degeneration are pain, weakness, hotness, shooting pains in the arms or legs (radicular pain), numbness and tingling in the extremities. The pain occurs during bending, lifting, or twisting. Weakness occurs in the leg muscles or foot drop and shows sign of damage to the nerve root. An injury to the lower back is known as a lumbar strain. Damaged tendons and muscles lead to feeling sore. The spinal cord is segmented into three levels,the spinal cord, spinal nerve and vertebral level. Segmentation of spinal cord is used for measuring spinal cord atrophy, which detects various disorders. The segmentation of the spinal cord is performed manually or automatic segmentation. Segmentation of the spine is performed in CT/MRI images. Segmentation of the spine in MR images helps with surgical planning by separating the vertebrae from the backdrop.

### 1.1 Challenges in DH Detection at Early Stages through MR Images

Detecting disc herniation (LDH) in magnetic resonance imaging (MRI) presents several challenges, particularly at early stages. These challenges and limitations of current imaging techniques in diagnostic technologies are: (i) resolution and contrast (ii) interpretation and challenges, and (iii) Time-Consuming Processes. DH manifests in different shapes, sizes, and orientations, making it hard for radiologists to identify them consistently. The various forms of DH, such as bulges, protrusions, extrusions, and sequestration, necessitate advanced imaging techniques and interpretation skills to accurately diagnose DH at an early stage. MR images are considered the gold standard for diagnosing LDH, but it has inherent limitations such as Resolution and Contrast: Early-stage herniations may not always be adequately visualized due to insufficient resolution or contrast between the disc material and surrounding tissues. (ii) Interpretation Challenges: The interpretation of MRI images often relies on subjective assessments by radiologists, which can lead to inconsistencies in diagnosis. Automated systems have been proposed but still require significant manual intervention and validation. (iii) Time-Consuming Processes: The traditional method of diagnosing LDH involves a lengthy review of numerous MRI slices, which is labor-intensive and can delay patient care. Recent advancements in artificial intelligence (AI) and deep learning are being explored to enhance the detection of DH at an earlier stage. Deep Learning Techniques such as YOLO (You Only Look Once) models have shown promise in automating the detection process. These models can analyze MRI images more quickly than human radiologists and may improve diagnostic accuracy when trained on extensive datasets.

### 1.2 Problem Statement

Inner and outer side of spinal cord is affected by a number of disorders. Inside spinal cord is damaged through fluid-filled cavities, blockage of blood supply, vitamin deficiency, multiple sclerosis and syphilis. Outside spinal cord is damaged through compression or injury on spinal cord [1]. X-rays, MRI, CT scan, myelography and electromyogram are medical techniques used for the diagnosis of problems in the spinal cord. Treatment for spinal cord disorders depends on the location and severity of the condition [2]. There are many treatments, such as occupational therapy, surgery and medications to solve spinal cord problems. However, detection DH [3] at an earlier stage is challenging due to Limited Directional Selectivity [4] problem and moreover below 2mm DH is not detected with computer-aided diagnosis software. Classification of stages in DH is difficult due to human intervention and prediction of the pfirrman grade for mild stage of DH is challenging for the radiologist. However, existing algorithms such as Transverse Dyadic Wavelet Transform (TDyWT) [5] and Adaptive Kernel Fuzzy C-Means Clustering Algorithm (AKFCM) poorly denoised the image. The above methods provide blurry images with low-edge quality. AKFCM provides poor-quality images with low accuracy. The above problems were solved through the optimized Denoising Convolutional Neural Network (DnCNN) [6] and Classification Algorithm.

### 1.3 Contributions

To detect the size of DH in the spinal cord, denoising the spinal cord image plays an important role. To solve the above problem, Po-DnCNN method is proposed for denoising the spinal MR images and helps to measure DH of below 2mm. Further, Hippopotamus optimization- Fast Hybrid Vision Transformer (Ho – FastViT) is proposed for the classification of stages in DH.

(i) To apply Patch based image augmentation in MR images of SPIDER dataset and generate the nucleus pulposus region projected images for different lengths such as 0.5/1/1.5/2 mm and used for the detection of the DH at an earlier stage.

(ii) To enhance the nucleus pulposus region, Po-DnCNN algorithm is proposed for the perspective projection of the nucleus pulposus region in the spinal cord and enhance the vertebrae.

(iii) To classify the DH stages, Ho – FastViT algorithm is proposed and classifies the different stages of DH such as (i) Degeneration (ii) Prolapse (iii) Extrusion (iv) Sequestration based on the Pfirrman grade values. The classification of the HD size of below 2mm *i.e*., 0.5/1.0/1.5 mm is performed based on the the projection of the nucleus pulposus region through the proposed Ho-FastViT algorithm.

(iv) To compare the proposed methods, such as Po-DnCNN with the existing algorithms, such as DWT, SWT, for the denoising and classification of the DH MR images and evaluate the edge, contrast and quality of the spinal cord abnormal and normal MR image through statistical methods.

(v) To compare the proposed Po-DnCNN algorithm performance in different spinal cord DH MR image dataset and evaluate the accuracy and sensitivity.

## 2 LITERATURE SURVEY

Automatic spinal cord segmentation is time-consuming and leads to fewer measurement errors. MR images of 22 sciatica patients with intervertebral disc herniation, 35 herniated and 97 normal discs images are analyzed with sparse optimization, for vertebrae segmentation and classification. Morphological segmentation of spinal region is used for the diagnosis of spinal cord problems [7]. The dynamic 6-DOF disc loading simulator is developed and used for the diagnosis of disc damage and herniations. The simulator has complex motion combinations and allows for the artificial lesions in the disc for analysis [8]. The degenerative cervical spine is generated using the FE model for the cervical disc herniation and simulated the spinal motion. Different Geometry is used for the simulation of the degeneration in clinical classification of degeneration cervical spine. Cervical disc displacement, annulus fiber and facet joint stress can be simulated using the FE model [9]. The retrospective study after surgery was analyzed for pain and patient satisfaction [10]. The symptomatic disc herniation and spinal tumor are simultaneously segmented in the MR image [11]. A retrospective radiographic study analyzes the effect of lumbar disc herniation on the kinetic motion of adjacent segments. Translational motion and angular vibration in the body affect disc herniation [12]. MR images are enhanced for high-level feature extraction and detect discs from the lumbar spine through Convolutional neural networks (CNN) [13]. Intervertebral disc therapeutic is the alternative to surgery for a symptomatic herniation in both the cervical and lumbar spine; prognosis is analyzed using MR images [14]. The surgical results are evaluated through Odom’s criteria for DH operated patients and termed as keyhole laminoforaminotomy. The anterior cervical approach is preferred in cervical disc herniation surgery and is suited for foraminal disc herniation and foraminal stenosis [15]. The Oswestry scale and VAS scale values are used for the Low-Back Pain Disability measurement [16].

Disc herniation in the spinal cord is the condition when the soft inner core of an intervertebral disc *i.e*., nucleus pulposus, projects through a tear in the tougher outer layer *i.e*., annulus fibrosus. The stages in Spinal disc herniation are (i) Degeneration (ii) Prolapse (iii) Extrusion and (iv) Sequestration. In degeneration, the spinal disc wears/weakens and still remains intact [17]. In the Prolapse stage, the nucleus push out the annulus, causing the disc to bulge. In the extrusion stage, nucleus is forced out through a small tear in the annulus. In the Sequestration stage, disc ruptures and the nucleus spills out from the disc center. The DH is measured in the MR images based on the length of the projection of the pulposus nucleus [18]. Based on the length of projection, DH is classified as the Mild stage (3mm), Moderate stage (3 to 5 mm) and severe stage (above 5mm). The minimum size of 2mm to 3mm is detectable using MR images, and a figure below 2 mm is poorly detectable in MR images. For earlier DH detection, novel algorithms need to be proposed. The detection of DH at an earlier stage prevents pain and numbness, which hamper daily activities, bladder or bowel dysfunction can be prevented.

Inpainting technique is used for filling in damaged, degraded, or missing regions in an image [19]. Inpainting techniques in medical imaging improve diagnostic accuracy by filling in gaps due to noise, artifacts, or incomplete data acquisition. Deep learning and neural networks-based inpainting techniques are Patch-Based Methods, Generative Adversarial Networks and Shape-Aware Masking. Inpainting techniques are used for testing the image processing algorithms, particularly in the context of evaluating methods for the detection, diagnosis, and classification of diseases. These techniques focus on assessing the performance of various image processing algorithms for earlier detection of diseases. Inpainting techniques have practical applications in various fields, such as Object Removal, Image Restoration and image Augmentation. In image augmentation, synthetic images are created using inpainting methods, which can be used for image enhancement and classification, and increase the training dataset [20].

A novel deep learning pipeline has been developed for vertebra labeling and segmentation in spinal computed tomography images [21], complemented by a deep learning harmonization approach for multi-vendor MRI to enable robust intervertebral disc segmentation [22]. Additionally, a two-stage deep learning methodology is used for the detection [23] and classification of cervical spine fractures, and a multi-scale hybrid attention convolutional neural network has been designed for the automatic segmentation of lumbar vertebrae from MRI [24-27].

### 2.1 Inferences from Literature Survey

Spinal cord MR images are denoised with different methods such as Discrete Wavelet Transform (DWT), Transverse Dyadic Wavelet Transform (TDyWT), and Stationary Wavelet Transform (SWT). Researchers segment the regions of the spinal cord through automatic or semiautomatic methods for precise visualization of spinal care regions with more accuracy. MR image has noise due to artifacts and need the appropriate image denoising techniques, to remove the blur and unclear orientation in images for accurate diagnosis of spinal cord diseases. DWT denoises the blur and ringing noise with poor edge enhanced in MR images and unsuitable for the DH diagnosis and other spinal disease diagnosis. SWT and DyDWT methods produce less accuracy due to poor edge enhancement of the spinal regions in the image. TDyWT produces better edges and never provides high accuracy due to discontinuity in edges. To solve the above problem, an optimized Denoising Convolutional Neural Network (DnCNN) is proposed. The proposed DnCNN method provides a clear edge, contrast and better quality than traditional methods.

## 3 METHODOLOGY

For the DH study, Spinal cord MR images are obtained from the Grand Challenge – SPIDER dataset consisting of Lumbar spine MR images of 257 patients with a resolution of 3.3 x 0.33 x 0.33 mm to 4.8 x 0.90 x 0.90 mm. Fig. (**1**) shows the block diagram of proposed Po-DnCNN based Enhancement and proposed HO – FastViT classification algorithm for DH stage classification. Initially, the MR image is preprocessed with DWT, DyDWT, SWT and TDyWT methods. Then, it is enhanced with the proposed Po-DnCNN algorithm and enhances the small variation in spine image, which is used for the analysis and prognosis of DH. To solve the problem of earlier detection of DH, MR images are enhanced with Parrot optimized DnCNN and enhance the small edges for high visual interpretation. Po-DnCNN enhances the nucleus pulposus region through optimized convolution layers. The proposed Po-DnCNN enhances the edges of the nucleus pulposus region, enhances the texture and detects the variations in the spinal cord image.

The Transverse Dyadic Wavelet Transform (TDyWT) has several advantages over traditional methods, such as improved Detail Extraction and noise Resistance. Moreover, efficient data representation through compact representation and adaptive processing is needed. TDyWT is used in Edge Detection, Damage Detection, Geometric Distortion Correction in images. TDyWT can be used for Fast Implementation through Parallel Processing Capabilities. Transverse Dyadic Wavelet Transform enhances the resolution, efficient data representation, versatility across various applications, and improved computational efficiency. DnCNN (Denoising Convolutional Neural Network) is a deep learning used in image denoising. DnCNN Improves Denoising Performance with Higher PSNR and SSIM and has the adaptability to Different Noise Levels. DnCNN has Enhanced Computational Efficiency through Reduced Processing Time and has efficient Memory Usage. DnCNN is robust to overfitting through Regularization Techniques. DnCNN implementations with pre-trained weights allow users to quickly apply the model without extensive training from scratch. DnCNN models provide enhanced denoising performance, improved computational efficiency, robustness against overfitting, versatility across applications, and user-friendly implementation options.

In medical image preprocessing, preprocessing plays a vital role in the analysis of MR images. The accuracy of segmentation is based on the effective preprocessed MR images. The pre-processing improves the MR image and suppresses undesired distortions. Image preprocessing is for precise information, extraction and accurate diagnosis. The preprocessing techniques, such as Image resizing, converting images from RGB to grayscale and image denoising, are performed with DWT, SWT and TyDWT. De-noising methods preserve the edges and remove noise in MR Images.

### 3.1 Denoising Convolutional Neural Network (DnCNN)

This is a type of deep learning model specifically designed for removing noise from images, particularly in medical imaging. It learns to differentiate between noise and actual image data, improving the clarity of images used for diagnosis.

### 3.2 Magnetic Resonance Imaging (MRI)

A non-invasive imaging technique used to visualize internal structures of the body, particularly soft tissues like the spinal cord and intervertebral discs. MRI is crucial for diagnosing conditions such as disc herniation.

### 3.3 Disc Herniation (DH)

This refers to a condition where the inner core of a spinal disc (nucleus pulposus) protrudes through a tear in the outer layer (annulus fibrosus). This can cause pain and neurological symptoms due to pressure on surrounding nerves.

### 3.4 Pfirrmann Grade

A classification system used to assess the severity of disc degeneration based on MRI findings. It helps in determining the stage of disc herniation, which is essential for treatment planning.

### 3.5 Parrot optimization tuned Denoising Convolutional Neural Network (Po-DnCNN)

The Po-DnCNN algorithm outperforms traditional denoising algorithms in terms of accuracy, image clarity, and the ability to detect smaller disc herniations effectively, making it a valuable tool in medical imaging for diagnosing disc-related conditions.

### 3.6 Hippopotamus Optimization - Fast Hybrid Vision Transformer (Ho-FastViT)

This is an advanced algorithm proposed in our study for classifying different stages of disc herniation based on enhanced imaging data. It combines optimization techniques with transformer architectures to improve classification accuracy.

### 3.7 Patch-Based Image Augmentation

A technique used to enhance training datasets by creating variations of existing images. This helps in improving the robustness of machine learning models by providing them with diverse examples.

### 3.8 Discrete Wavelet Transform (DWT)

A mathematical technique used for signal processing that helps in decomposing images into different frequency components, often employed in image denoising.

### 3.9 Stationary Wavelet Transform (SWT)

Similar to DWT it maintains shift invariance, making it useful for analyzing images with varying structures without losing information about their location.

### 3.10 Inpainting

A method used to fill in missing or damaged areas of an image, improving overall image quality and aiding in accurate diagnosis by restoring lost details.

### 3.11 Segmentation

The process of partitioning an image into multiple segments or regions to simplify its representation and make it easier to analyze specific structures, such as vertebrae or discs in spinal images.

### 3.12 Nucleus Pulposus

The soft inner core of an intervertebral disc that can herniate and cause pain when it protrudes through the outer layer.

### 3.13 Annulus Fibrosus

The tough outer layer of an intervertebral disc that surrounds the nucleus pulposus and helps maintain the structural integrity of the disc.

### 3.14 Preprocessing of MR Images for Noise Removal

Noise removal plays a vital role in MR images for anatomical structure extraction. MR images are added with noises during image acquisition. MR Image is denoised with algorithms such as DWT, SWT, and TyDWT, removes the additive noise, and retains the maximum information in the MR image due to the retention of Low frequency components. Noise free MR image is required for accurate diagnosis because the noise degrades the edges of objects in the image. Denoising recovers the anatomical features in the MR image. Wavelet transform has localization property and removes the noise in the vertebrae and projects the nucleus pulposus region. Existing methods denoise the low and high frequency components separately and remove the pixels abruptly. To overcome this problem, DWT method is used and solves the above problem. DWT extracts the low and high frequency components using denoising filters. Low frequency component has high information on projected nucleus pulposus region and high-frequency components has high information on the vertebrae. Noise suppresses the high frequency components and leads to wrong diagnosis in DH. DWT denoises the MR image of spine and increases the contrast. Fig. (**2**) shows the threshold of spine images using Bayesian Threshold based DWT.

Soft and hard thresholding are applied in the spinal MR image. Hard thresholding provides the details in the image and soft thresholding provides the approximate value. The soft and hard thresholding is applied in the MR image and obtains the horizontal, vertical and diagonal components. The thresholding of wavelet coefficients is performed using the Bayesian method. Thresholding suppresses the noise in MR images. Many thresholding methods are available for wavelet coefficients. Identification of appropriate thresholding methods is challenging. Denoising and exaction of pixels are based on the threshold method. Bayesian Threshold is applied to MR images and removes the noises in the MR images.

The SWT based MR image denoising improves the visual information of degraded disc in the image. SWT overcomes the lack of translation-invariance of the discrete wavelet transform and denoises the vertebrae region. SWTs increase the accuracy of spinal cord disease diagnosis and improve image interpretation of spinal cord regions. Texture in the images is improved after the SWT preprocessing. SWT shows better textural features in the image. Fig. (**3**) shows the SWT denoising with Bayesian thresholding.

In Table 1, two patients’ SWT based Bayesian thresholding details, such as horizontal, vertical and diagonal for spinal cord, are shown for MR image levels. The horizontal detail reduces and increases the threshold for vertical and diagonal values in level 1 and details are similar in other thresholding levels.

The property of time invariance in SWT provides the high-frequency content edge details. The approximations are in high-scale and low frequency components, whereas details are in low-scale and high frequency components. SWT generates gradient images and low frequency components at different levels of MR image decomposition are eliminated. Discrete Wavelet Transform is not a time invariant and hence translation invariance is average in DWT, whereas in SWT, the threshold increases the similarity index.

TDyWT is applied in the MR image to denoise the DH regions. The transverse dyadic wavelet transforms (TDyWT) analyse the image with respect to spatial and frequency of pixels. TyDWT captures directional information in images and filters the structures with inherent orientation, edges and textures. TDyWT is based on low computations compared to wavelet transforms. The framework of TDyWT is shown in the Fig. (**4**). In TDyWT, downsampling reduces the dimensionality of the data, provides significant features and discards redundant pixels.

TDyWT improves the filter process based on directional features and achieves efficient multiresolution. TDyWT remains a valuable method for enhancing the vertebrae and projects the nucleus pulposus for higher visualization when compared to DWT and SWT. Fig. (**5**) shows the comparison of filters for MR images.

### 3.15 Po-DnCNN and TDyWT-DnCNN for Perspective Enhancement of Vertebrae and Projected Nucleus Pulposus Region

Parrot Optimization (Po) -Denoising Convolutional Neural Network methods enhance the nucleus region using residual learning and perspectival projects the vertebrae and projects DH regions. In this paper, residual learning and batch normalization speed up the training process, improve the image enhancement and increase the denoising performance. Po tunes the DnCNN layers, such as convolution layers and Batch normalization layers. In the convolution layers of DnCNN, (i) Dialation factor [1, 1] (ii) Weights (iii) Bias is tuned with Po. In Batch normalization of DnCNN(i) Scale (ii) Epsilon (iii) Trained Mean are tuned with Po. Parrot Optimization Algorithm (Po) is based on the behaviors of trained Pyrrhura Molinae parrots. Po mimics the social dynamics and behaviors of parrots, *i.e*., communication and foraging strategies. Po explores and exploits the solution space. Po algorithm balances exploration *i.e*., search for new potential solutions and exploitation, refines the known good solutions and avoids local optima and ensures a thorough search of the solution space. The algorithm exhibits adaptability under varying configurations and maintains the performance across different optimization scenarios. DnCNN enhances the image after reducing the additive white Gaussian noise. DnCNN uses residual learning and batch normalization and enhances lower frequency components in the image. Residual Learning simplifies the learning process based on the noise component rather than the entire image. Fig. (**6**) shows the architecture of the Po-DnCNN for perspective enhancement.

In DnCNN, Batch Normalization stabilizes the learning process through the normalizing of the outputs of each layer, which helps to convergence and reduces the internal covariate shift, makes the image perspective enhancement of the low frequency components. For DH detection, Po optimizes the Batch normalization and enhances the vertebrae and nucleus pulposus. The depth of the network is varied with Po optimized Convolution layers in the DnCNN, and captures complex features in the images. PO- DnCNN's architecture enhances the resolution of images by predicting high-frequency details. The pseudocode for Po-DnCNN is as below. Fig. (**7**) shows the perspective projection of the inpainted nucleus pulposus region using the proposed Po-DnCNN.

### 3.16 Classification of DH Stages using Ho – Fastvit Deep Learning Model

Hippopotamus Optimization (HO) algorithm is based on the behaviors of hippopotamuses. Features and advantages of the HO algorithm are the (i) Trinary-Phase Model, (ii) Position Updating, (iii) Defensive Strategies, and (iv) Evasion Methods. The HO algorithm is structured using the trinary-phase model, which updates the position in rivers or ponds. The algorithms include the reflecting nature of the hippopotamuses against predators. In HO, the escape methods for threats are used. The Ho algorithm has impressive results and ranks first in 115 out of 161 benchmark functions, such as unimodal and high-dimensional multimodal functions. The HO algorithm balances exploration *i.e*., searches for new areas and exploitation, refines the known good solutions, avoids local optima and ensures a thorough search of the solution space. The HO algorithm solves complex, nonlinear, and high-dimensional optimization problems. Benchmark Testing is done based on multiple benchmark functions, such as CEC 2019 and CEC 2014 test suites, and shows robustness and effectiveness.

FastViT is a hybrid vision transformer model that integrates convolutional neural networks (CNNs) and transformers. FastViT is used for the fast classification of images in computer vision. FastViT combines the feature extraction capabilities of CNNs with the attention mechanisms of transformers captures global context for image classification. FastViT has Structural Reparameterization, which optimizes the model's structure by removing the unnecessary skip connections, reduces memory access costs and enhances computational efficiency. The RepMixer Operator in FastViT consists of a token mixing operator called RepMixer, which improves the model's performance through facilitating effective information flow without incurring high latency. FastViT operates 3.5 times faster than CMT (a state-of-the-art hybrid transformer) and 4.9 times faster than EfficientNet, and maintains comparable accuracy. Ho-FastViT is precise in localization and classification. Ho-FastViT shows resilience against out-of-distribution. Ho-FastViT has a compelling balance between speed and accuracy. Fig. (**8**) shows the architecture of the Ho-FastViT for classification. Pseudocode for proposed Ho- FastViT is shown below.

## 4 RESULTS AND DISCUSSION

### 4.1 DH Image Filter – Po-DnCNN

In Table **2**, the proposed Po-DnCNN method has better accuracy and high value than existing methods for abnormal spinal cord images. Tables **3**-**5** show Po-DnCNN performance through the quality, edge and contrast values of the normal and abnormal DH spinal cord image. Compared to other statistical methods, the proposed Po-DnCNN method has high performance.

In Table **3**, the proposed Po-DnCNN method has better contrast, accuracy and high value than other statistical methods for normal and abnormal DH spinal cord MR image

In Table **4**, the proposed Po-DnCNN method has enhanced edges and accuracy, which is proved through normal and abnormal spinal cord image statistical values.

In Table **4**, the proposed Po-DnCNN method has enhanced edge, accuracy and proved through the normal and abnormal DH spinal cord image statistical values. In Tables **2**-**4**, DWT, SWT, and TDyWT method has some disadvantages, such as low-quality MR images are not suitable for analysis, poorly discovering non-convex shape, and never performing for the variable illumination levels in MR images. The proposed Po- DnCNN method overcomes the above disadvantages, gives high statistical values for DH spinal cord image, and shows enhanced DH spinal cord image.

### 4.2 Classification of DH type - Ho – Fastvit

Tables **5** and **6** show the performance of the proposed Ho – Fastvit classification algorithm and the statistical values of DH spinal cord image. DH spinal cord image performance measures such as precision, F-measure (%), specificity, BCR, BER (%), F-measure of sens / spec (%), geometry accuracy, pF measure (%), NRM, PSNR, DRD and MPM (*1000) are analysed for the abnormal and normal DH image.

The average of these values for the above parameter is close to unity (100%) and proves quantitatively that the proposed Ho – Fastvit classifies the DH spinal cord image. Existing method provides high value of precision and recall compared to the Ho – Fastvit proposed method. However, both these methods produce visually low-quality output. Ho – Fastvit proposed methods show better visual quality images. Among these, the proposed Ho – Fastvit method achieves high value of precision, recall and other parameters for normal and abnormal images. As shown in Table **6**, the proposed Ho – Fastvit method has better accuracy and high parameter value than other statistical methods for normal spinal cord MR image.

### 4.3 Prediction of DH Type and Pfirrman Grade

In Disc degeneration, the disc has less water content and the annulus fibrosus becomes weak. Degeneration reduces the disc height. In Prolapse, nucleus pulposus bulges or protrudes through the annulus fibrosus. This is known as a disc prolapse or herniation. In Extrusion, the nucleus pulposus ruptures through the annulus fibrosus, and the outer layer of the annulus remains intact. In Sequestration, complete separation of the extruded disc material from the parent disc is required. The progression through these stages is not always linear or predictable. Disc degeneration is a complex process influenced by various factors like age, genetics, injury and lifestyle. The DH classification is performed using the proposed Ho – FastViT method. Fig. (**9**) shows the comparison of the proposed algorithm with existing algorithms.

The Pfirrmann grading system assesses the degeneration of lumbar intervertebral discs based on MR images. Pfirrmann grades are based on specific characteristics of the disc's appearance and structure. In Grade I, the disc appears homogeneous with a bright hyperintense white signal intensity and maintains normal disc height. Grade II is for inhomogeneous and exhibits a hyperintense white signal. The distinction between the nucleus pulposus and annulus fibrosus is clear, and the disc height remains normal. In Grade III, the disc shows an inhomogeneous structure with an intermittent gray signal intensity. There is a loss of clear differentiation between the nucleus and annulus, and the disc height is normal or slightly decreased. In Grade IV: The disc appears inhomogeneous with a hypointense dark gray signal intensity. There is no distinction between the nucleus and annulus, and the disc height is moderately decreased. In Grade V, the disc is inhomogeneous with a hypointense black signal intensity, indicating the complete collapse of the disc space and no differentiation between the nucleus and annulus. Table **7** shows the prediction of Pfirrmann Grading Accuracy using Proposed Ho-FastViT method.

### 4.4 Ablation Study in PO-DNCNN

An ablation study is a systematic method used for the evaluation of the contribution of different components or configurations within a model. Ablation studies help researchers to understand how various architectural choices and hyperparameters affect the performance of the network in image denoising tasks [28]. Ablation studies in DnCNN can be performed in the (i) Component Analysis, (ii) Layer Contribution (iii) Activation Functions (iv) Hyperparameter Tuning [29] (v) Learning Rate and Batch Size (vi) Kernel Sizes (vii) Noise Level Variability Training on Different Noise Levels and (viii) Data Augmentation Techniques. Benefits of conducting an ablation study are model optimization, enhanced interpretability and guided future research. Ablation studies [30] in DnCNN play a crucial role in optimizing and understanding deep learning models [31] for DH MR image denoising. Fig. (**10**) shows the performance of the Po-DnCNN after ablation study in the Hyperparameter – Accuracy. Fig. (**11**) shows the performance of the Po-DnCNN after ablation study in the Activity Function. Fig. (**12**) shows the performance of the Po-DnCNN after ablation study in the Activity Function. Fig. (**13**) shows the performance of the Po-DnCNN and different wavelet transforms denoising.

## 5 DISCUSSION

Po-DnCNN in the context of disc herniation imaging led to a significant advancement in medical image processing [32]. This discussion explores the methodology, advantages, challenges, and implications of using Po-DnCNN for denoising MRI scans of disc herniations [33]. High-resolution MRI scans are critical for diagnosing disc herniation. However, these images often contain noise due to various factors such as patient movement, magnetic field inhomogeneities, and acquisition parameters. Po-DnCNN architecture consists of convolutional layers, batch normalization, and ReLU activation functions. The network is designed to learn residuals between noisy and clean images, enabling effective noise reduction. The model is optimized with Po using a loss function that minimizes the difference between the predicted clean image and the actual clean image. This process requires careful tuning of hyperparameters such as learning rate and batch size. Po-DnCNN improved Diagnostic Accuracy, Preserves Structural Details: Unlike traditional denoising methods that may blur important anatomical structures, DnCNN maintains edge sharpness and structural integrity, which is crucial for accurate diagnosis. By providing clearer images through Po-DnCNN, radiologists improve in decision-making processes P0-DnCNN for denoising MRI scans in the context of disc herniation presents a promising avenue for improving diagnostic accuracy and patient care. While challenges related to data requirements and computational resources exist, the potential benefits in terms of enhanced image quality and adaptability make it suitable for earlier detection of DH. Fig. (**14**) shows the performance of the Po-DnCNN for Different Dataset.

Table **8** shows the comparison of the proposed algorithm with the existing algorithm.

## CONCLUSION

In this paper, the proposed Po-DnCNN enhances DH spinal cord image. Compared to Exiting methods method, the proposed Po-DnCNN has better perspective enhanced region of vertebrae and DH region. Po-DnCNN method gives satisfactory performance through enhanced and clear structure with discontinuities in boundary regions compared to existing methods. Po-DnCNN method preserves the shape, sharp edge and better visual quality. The spinal cord has high visualisation after processing with the proposed Po- DnCNN method and enhanced the nucleus pulposus and vertebrae through the different training functions. Traditional algorithms such as Transverse Dyadic Wavelet Transform (TDyWT) perform less during denoising due to irregular shape, size, colour and texture features in DH spinal cord region in MR images. The statistical parameter of normal and abnormal spinal cord images shows the enhanced image features such as edges, contrast and quality through Po-DnCNN and provides a high accuracy of detection of about 98%, compared to traditional methods. The training function provides a perspective view of the spinal cord. Ho- Fastvit method classifies the stages of DH with high accuracy of about 97% compared to traditional methods after processing the image with the proposed Po-DnCNN algorithm.

## Figures and Tables

**Fig. (1):**
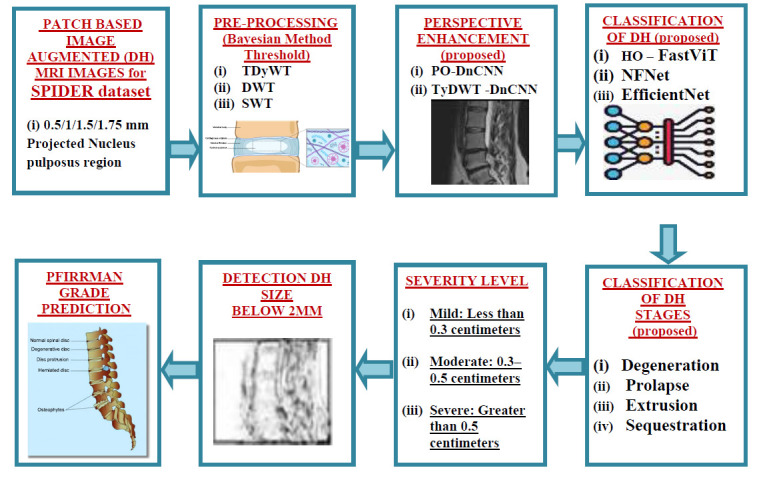
The block diagram of DNCNN for spine image.

**Fig. (2):**
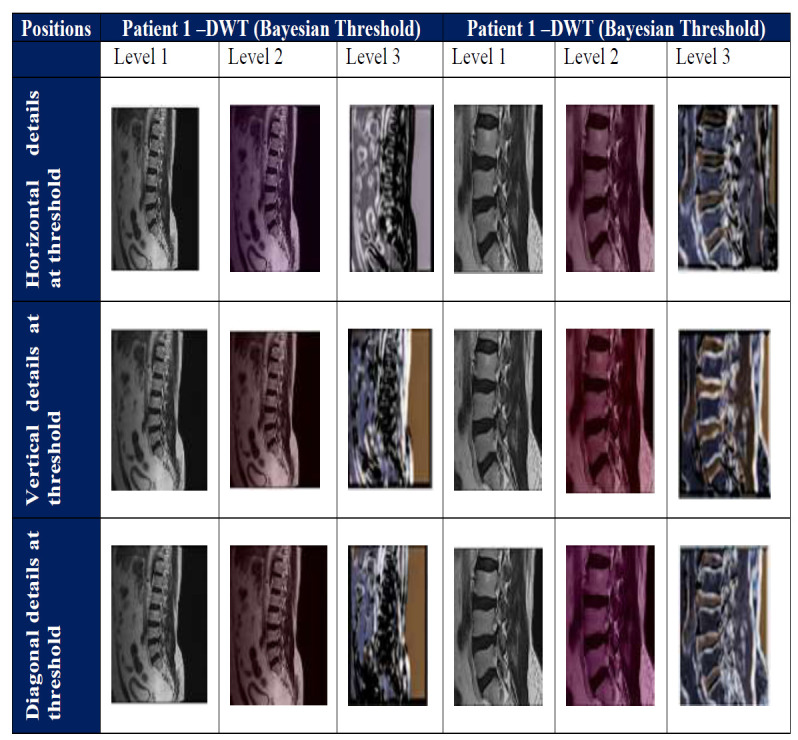
Bayesian threshold of spine images for DWT algorithm.

**Fig. (3):**
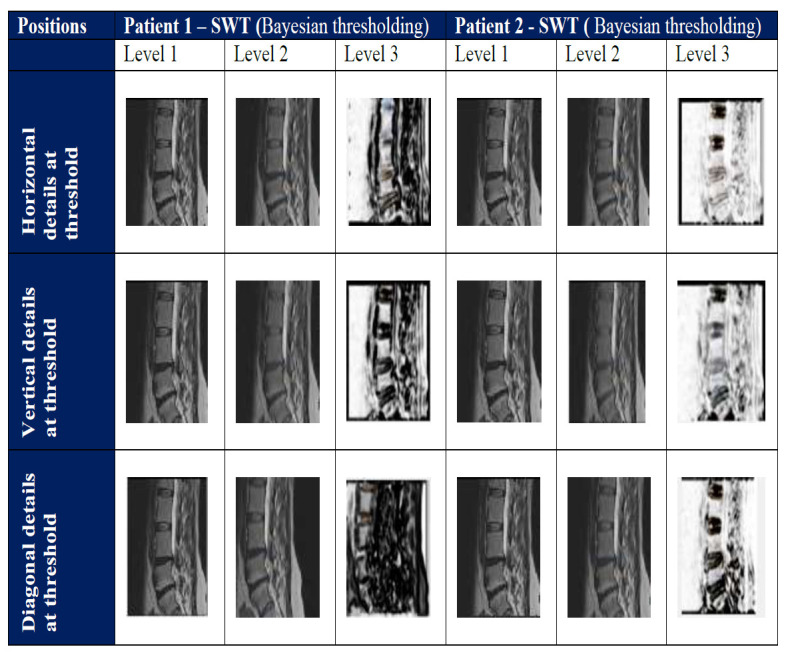
Threshold of spine images for SWT algorithm.

**Fig. (4):**
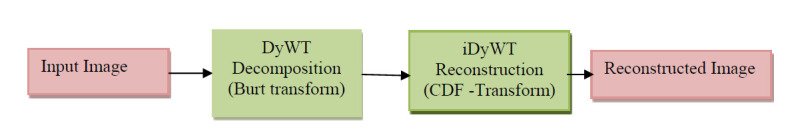
Framework of the proposed TDyWT.

**Fig. (5):**
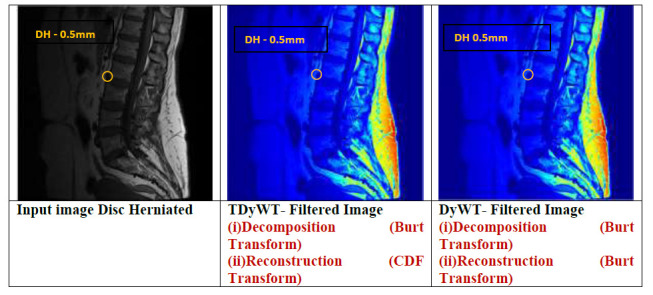
Comparison of TDyWT and DyWT filter.

**Fig. (6):**
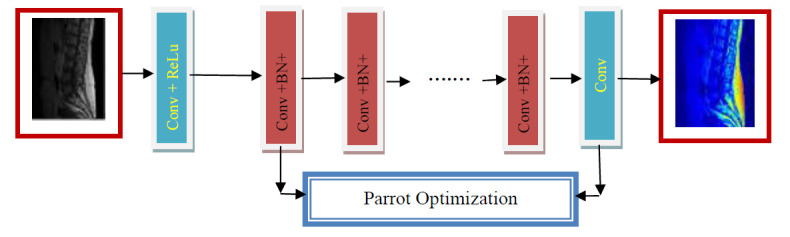
Architecture of the Po-DnCNN for perspective enhancement.

**Fig. (7):**
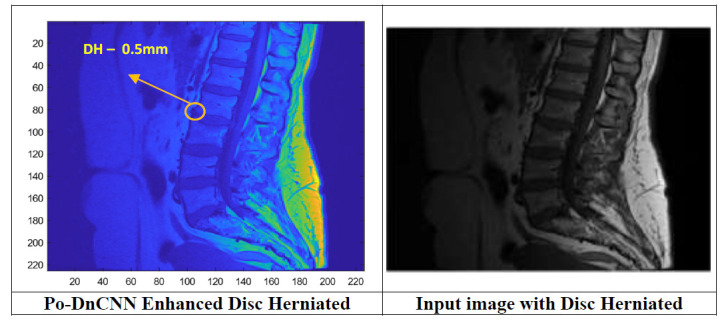
Perspective projection of the inpainted nucleus pulposus region using proposed Po-DnCNN.

**Fig. (8):**
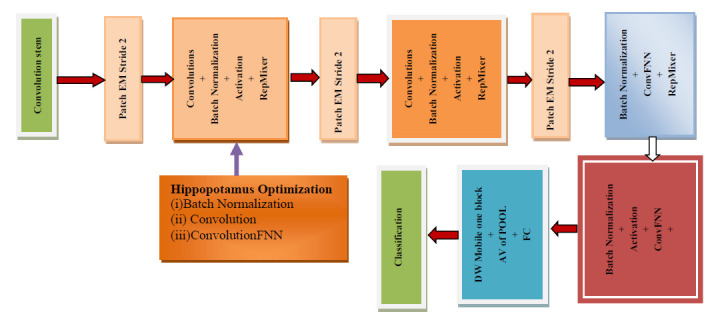
Proposed architecture of the Ho-FastViT for classification.

**Fig. (9):**
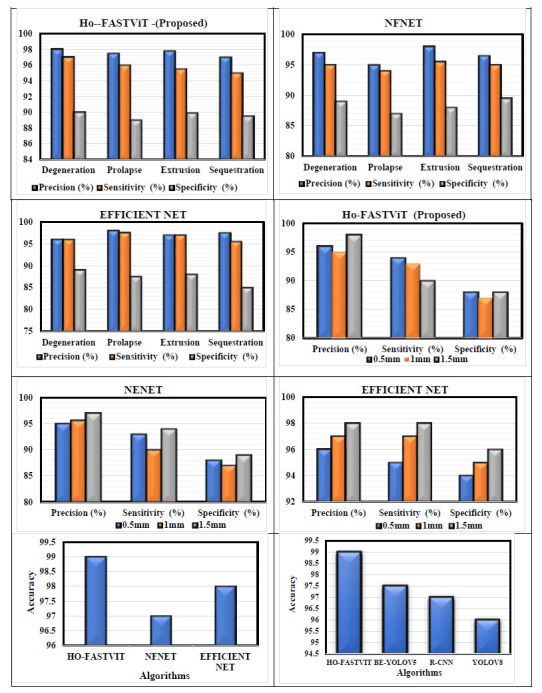
Comparison of the proposed Ho-FastViT algorithm with exiting algorithms for classification of DH.

**Fig. (10):**
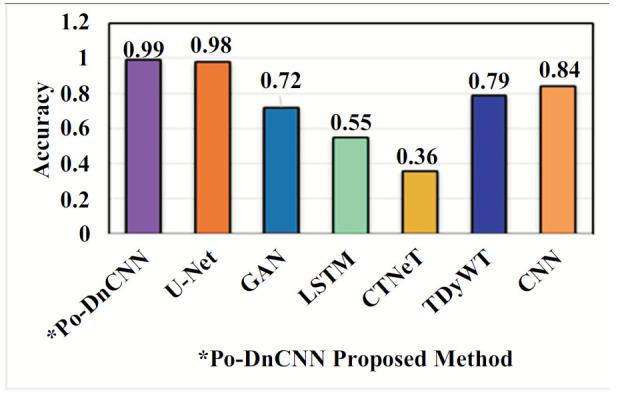
Performance of the Po-DnCNN after ablation study in the hyperparameter - accuracy.

**Fig. (11):**
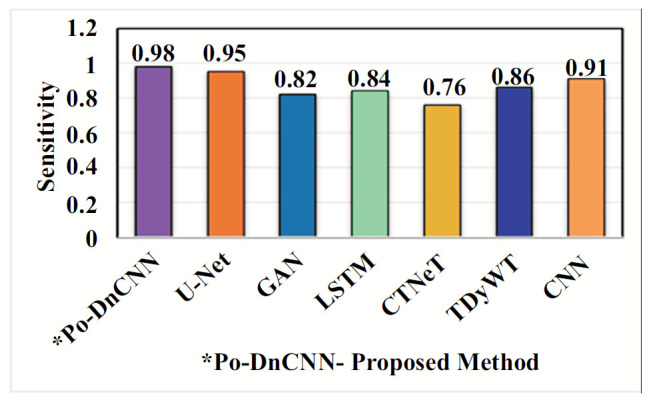
Performance of the Po-DnCNN after ablation study in the activity function.

**Fig. (12):**
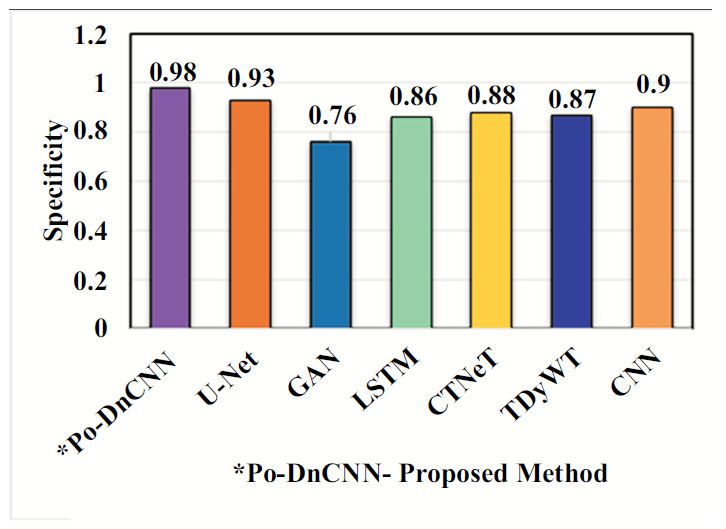
Shows performance of the Po-DnCNN after ablation study in the activity function.

**Fig. (13):**
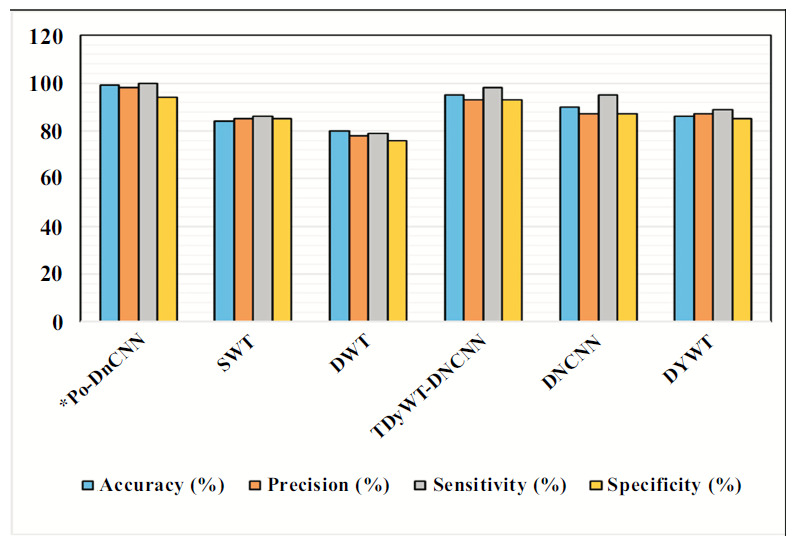
Performance of the Po-DnCNN and different wavelet transforms denoising.

**Fig. (14):**
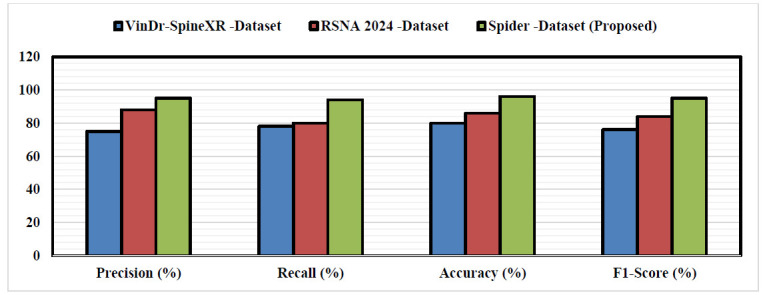
Performance of the Po-DnCNN for different dataset.

**Table 1:** Detail of patient – 1 and patient 2 at threshold (SWT).

Levels	Patient 1 – SWT (threshold)			Patient 2 – SWT (threshold)		
	Horizontal	Vertical	Diagonal	Horizontal	Vertical	Diagonal
**Level 1**	671.2	286.5	372.9	1116	896.9	729.6
**Level 2**	301.2	159.1	156.5	561.6	447.2	336.3
**Level 3**	137.7	84.76	83.47	280.4	240	180.3
**Level 4**	93.86	36.36	45.46	177.2	110.1	90.75
**Level 5**	60.47	18.52	20.15	110.7	60.79	39.07

**Table:** 

Pseudocode for proposed Po-DnCNN
***# Initialize parameters*** ***population_size = N*** ***max_iterations = T*** ***dimension = D*** ***population = initialize_population(population_size, dimension)*** ***# Evaluate fitness of each parrot in the population*** ***for each parrot in population:*** ***parrot.fitness = evaluate_fitness(parrot)*** ***# Main optimization loop*** ***for iteration in range(max_iterations):*** ***# Identify the leader parrot based on fitness*** ***leader = find_leader(population)*** ***# Update positions of each parrot*** ***for each parrot in population:*** ***if parrot is leader:*** ***# Leader behavior: move towards the best-known position*** ***parrot.position = update_leader_position(parrot, leader)*** ***else:*** ***# Follower behavior: move towards the leader with some exploration*** ***parrot.position = update_follower_position(parrot, leader)*** ***# Evaluate the fitness of the updated position*** ***parrot.fitness = evaluate_fitness(parrot)*** ***# Optionally apply DnCNN for denoising on the parrot positions*** ***Enhanced_image = DnCNN(parrot.position)*** ***# Return the best solution found*** ***best_solution = find_best_solution(population)*** ***return best_solution***

**Table:** 

Pseudocode for proposed Ho- FastViT
**# Initialize population of hippopotamus individuals** **population = initialize_population(size)** **# Evaluate fitness of each hippopotamus** **for hippo in population:** **hippo.fitness = evaluate_fitness(hippo)** **# Main optimization loop** **while not termination_criterion():** **# Identify the leader hippopotamus** **leader = find_leader(population)** **# Update positions of each hippopotamus** **for hippo in population:** **if hippo is leader:** **# Leader behavior: move towards the best-known position** **hippo.position = update_leader_position(hippo, leader)** **else:** **# Follower behavior: move towards the leader with some exploration** **hippo.position = update_follower_position(hippo, leader)** **# Evaluate the fitness of the updated position** **hippo.fitness = evaluate_fitness(hippo)** **# Apply FastViT for image processing on the hippopotamus positions** **processed_image = FastViT(hippo.position)** **# Return the best solution found** **best_solution = find_best_solution(population)** **return best_solution**

**Table 2:** Contrast values for normal spinal cord image after Po-DnCNN denoised with traingdx function.

**Image ID**	**DWT**	**SWT**	**TDyWT**	**AKFCM**	**TDyWT-DnCNN** **(Proposed)**	**Po-DnCNN** **(Proposed)**
**MSN0011**	112	143	123	122	132	181
**MSN0097**	111	160	134	131	117	183
**MSN0023**	118	175	149	149	112	185
**ITA0005**	108	236	112	106	169	186
**MSA0069**	109	161	121	120	120	187
**MSA0009**	100	198	120	117	110	188

**Table 3:** Edge for abnormal DH spinal cord image after Po-DnCNN denoised with traingdx function.

**Image ID**	**DWT**	**SWT**	**TDyWT**	**AKFCM**	**TDyWT-DnCNN (Proposed)**	**Po-DnCNN (Proposed)**
**MSN0011**	124	126	119	114	109	87
**MSN0097**	125	123	113	109	110	90
**MSN0023**	127	118	110	106	113	88
**ITA0005**	98	67	109	105	81	87
**MSA0069**	126	123	117	113	120	89
**MSA0009**	124	127	121	116	121	88

**Table 4:** Visual quality-entropy for abnormal DH spinal cord image after Po-DnCNN denoised with traingdx function.

**Image ID**	**DWT**	**SWT**	**TDyWT**	**AKFCM**	**TDyWT-DnCNN (Proposed)**	**Po-DnCNN (Proposed)**
**MSN0011**	125	126	107	109	99	85
**MSN0097**	126	127	102	111	94	86
**MSN0023**	127	128	106	104	96	88
**ITA0005**	98	99	104	102	100	89
**MSA0069**	126	127	105	106	98	87
**MSA0009**	124	125	101	105	95	84

**Table 5:** Performance of Normal spinal cord image.

**Parameters**	**LSTM**	**SVM**	**CNN**	**EfficientNet (Proposed)**	**NFNet (Proposed)**	**Ho – Fastvit (Proposed)**
**Precision (%)**	96	92	75	77	96	93
**F-measure (%)**	97	96	85	87	98	97
**Specificity (%)**	99	99	95	91	99	95
**BCR (%)**	99	97	98	95	97	98
**BER (%)**	47	32	45	68	25	54
**F-measure of sens / spec (%)**	99	98	97	96	99	99
**Geometry accuracy (%)**	99	99	98	96	99	97
**pF measure (%)**	97	96	86	88	99	96
**NRM**	0.005	0.004	0.03	0.05	0.003	0.03
**PSNR**	22	23	14	12	24	14
**DRD**	2	1	7	18	8	16
MPM(*1000)	0.2	2	1	4	0.02	2

**Table 6:** Performance of abnormal DH spinal cord MR image.

**Parameters**	**LSTM**	**SVM**	**CNN**	**EfficientNet (Proposed)**	**NFNet (Proposed)**	**Ho – Fastvit (Proposed)**
**Precision (%)**	97	93	77	79	97	94
**F-measure (%)**	99	97	89	90	97	98
**Specificity (%)**	99	98	93	94	97	96
**BCR (%)**	99	96	97	94	95	99
**BER (%)**	50	40	50	70	30	74
**F-measure of sens / spec (%)**	99	98	96	97	99	97
**Geometry accuracy (%)**	99	98	97	96	98	96
**pF measure (%)**	98	97	87	89	99	97
**NRM**	0.006	0.005	0.03	0.07	0.004	0.06
**PSNR**	24	25	17	16	28	15
**DRD**	3	2	8	19	9	17
MPM (*1000)	0.3	3	2	5	0.03	3

**Table 7:** Prediction of Pfirrmann grading accuracy using Proposed Ho-FastViT method.

**Patient -ID**	**Pfirrmann Grading** **(Laboratory Measured)**	**Pfirrmann Grading** **(Predicted Measured)** **Ho-FastViT [Proposed]**	**BE-YOLOV5** **(Ref No:25)**	**YOLOV8** **(Ref No:26)**	**R-CNN** **(Ref No:27)**
**P-98**	**5**	**5**	**5**	**5**	**5**
**P-177**	**4**	**4**	**4**	**4**	**5**
**P-45**	**3**	**2**	**3**	**4**	**3**
**P-78**	**5**	**5**	**4**	**4**	**4**
**P-107**	**5**	**5**	**4**	**5**	**4**
**P-50**	**4**	**4**	**5**	**5**	**4**

**Table 8:** Comparison of the proposed algorithm with existing algorithms.

**Algorithm/Refs.**	**Accuracy (%)**	**Precision (%)**	**Recall (%)**	**F1-Score (%)**	**Unique Features**
Preprocessing Techniques in Deep Learning [34]	85.4	82.3	80.7	81.5	Focus on noise reduction and data enhancement before classification.
MRI Imaging for Hyperostosis Frontalis Interna (HFI) [35]	88.2	86.5	84.2	85.3	Specialized in enhancing MRI visualization of skeletal anomalies.
Multimodal Segmentation of Bone Metastasis [36]	90.1	89.0	87.5	88.2	Multimodal data fusion for enhanced lesion segmentation accuracy.
Automated Knee Joint Localization [37]	89.6	88.4	87.0	87.7	Fast and accurate localization, optimized for musculoskeletal imaging.
**Proposed Algorithm (Po-DnCNN)**	98.0	97.0	96.89	96.0	Enhanced denoising with optimized feature extraction for improved accuracy.

## Data Availability

The authors confirm that the data supporting the findings of this research are available within the article.

## LIST OF ABBREVIATIONS

MRI: Magnetic resonance imaging
DWT: Discrete Wavelet Transform
SWT: Stationary Wavelet Transform
Ho–FastViT: Hippopotamus optimization- Fast Hybrid Vision Transformer
Po-DnCNN: Parrot optimization tuned Denoising Convolutional Neural Network
DH: Disc Herniation
